# Supplementary material for: The G-Protein-Coupled Estrogen Receptor Agonist G-1 Mediates Antitumor Effects by Activating Apoptosis Pathways and Regulating Migration and Invasion in Cervical Cancer Cells
Source: Cancers (Basel). 2024 Sep 27;16(19):3292. doi: 10.3390/cancers16193292 (PMC11475807; doi:10.3390/cancers16193292)
Supplement: Supplementary file 1 [file cancers-16-03292-s001.zip › cancers-3203902-supplementary.pdf]

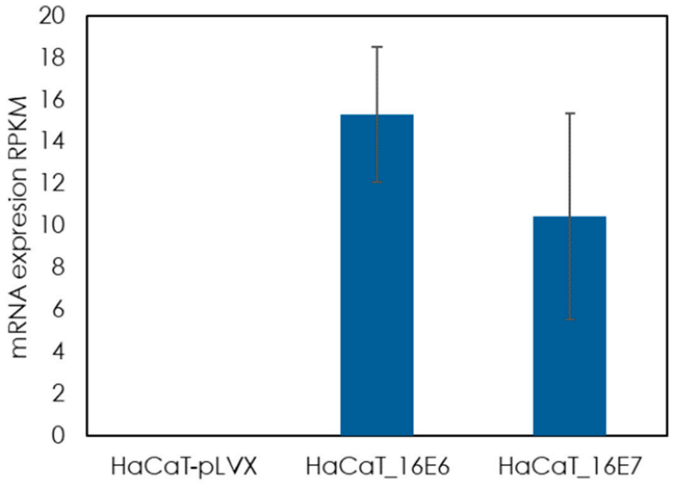

Supplemental Figure S1. Comparison of reads per kilobase per million mapped reads (RPKM). Values for E6 and E7 oncogenes expression were measured by RNA-Seq data from HaCaT transduced cell lines using HaCaT-pLVX, HaCaT-16E6, and HaCaT-16E7.

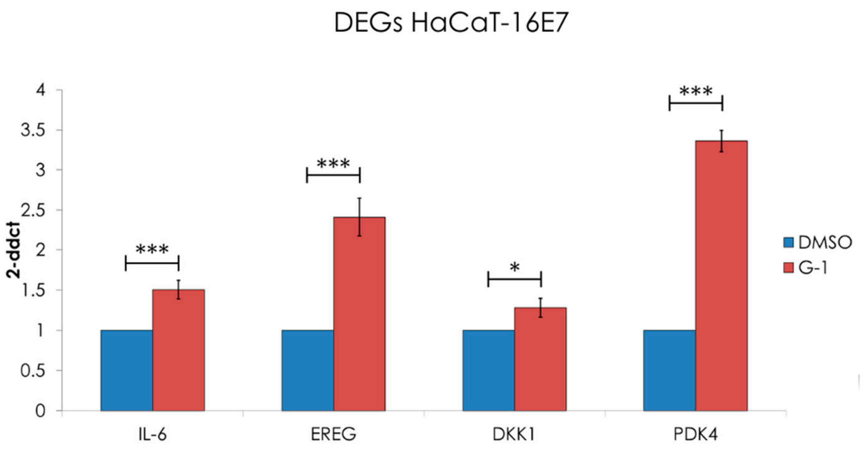

Supplemental Figure S2. Identification of differentially expressed genes by G-1. Relative mRNA expression of upregulated genes by qPCR. RPLP0 was used as a reference gene. Statistical significance (* P≤ 0.05; ** P ≤ 0.01; *** P ≤ 0.001).
